# Human Pose Estimation for Real-World Deployment: A Review of Methods, Systems, and Applications

**DOI:** 10.3390/s26154808

**Published:** 2026-07-28

**Authors:** Hyun-Ae Lee, Zheyu Zhang, Seong-Yoon Shin

**Affiliations:** Department of Computer Science and Information Engineering, Kunsan National University, Gunsan 54150, Republic of Korea; tranhyun@gmail.com

**Keywords:** human pose estimation, real-world deployment, benchmark-to-deployment gap, intelligent sensors, edge computing, system pipeline, model efficiency, temporal stability, deployment-oriented evaluation, application reliability

## Abstract

Human pose estimation (HPE) has become a core technology for human-centered visual understanding and is widely used in sports analysis, rehabilitation assessment, human–computer interaction, autonomous driving, and industrial safety monitoring. Although benchmark-driven research has substantially improved pose estimation accuracy, strong performance on standard datasets does not necessarily ensure reliable deployment in real-world environments. Practical HPE systems are commonly deployed in camera-based, mobile, wearable, and edge-computing environments. In these settings, sensor quality, device placement, frame rate, illumination conditions, hardware limitations, temporal instability, and application-specific reliability requirements jointly influence system performance. Using a structured narrative synthesis of representative literature, this review examines HPE from a deployment-oriented perspective instead of treating pose estimation solely as a model-level task. The review first summarizes the major benchmark-to-deployment gaps, including data, system, task, and temporal gaps; it then analyzes the end-to-end HPE pipeline, including image or video acquisition, human detection, region localization, pose inference, tracking, temporal smoothing, coordinate decoding, and task-level decision-making. Representative CNN-based, Transformer-based, temporal modeling, and lightweight optimization methods are reviewed in terms of deployment cost, optimization potential, and application suitability. This review also discusses practical application requirements and future research directions related to deployment-aware evaluation, system-level optimization, efficient temporal modeling, and application-level reliability. By linking benchmark performance with practical sensing and system constraints, this review provides a structured reference for the selection, evaluation, and deployment of HPE methods in real-world environments.

## 1. Introduction

Human pose estimation (HPE) is a core task in human-centered visual understanding because it converts human images and videos into structured representations of joints, limbs, and motion. HPE supports applications in sports analytics, rehabilitation assessment, surveillance, human–computer interaction, autonomous driving, and industrial safety monitoring. Over the past decade, HPE has evolved from handcrafted and shallow-learning pipelines to deep-learning frameworks for 2D pose estimation, 3D pose estimation, multi-person pose estimation, and video-based pose tracking. This progress has been supported by benchmark datasets and standardized evaluation protocols, including COCO, MPII, Human3.6M, and PoseTrack [1,2,3,4].

Benchmark-oriented research has produced substantial methodological advances. CNN-based models such as SimpleBaseline and HRNet improved keypoint localization through heatmap regression and high-resolution feature representations [5,6]. Transformer-based methods such as ViTPose further improved benchmark accuracy by strengthening global dependency modeling [7]. Real-time and lightweight systems, including RTMPose, Lite-HRNet, and PoseMamba, indicate a growing focus on efficiency, temporal modeling, and deployment-oriented design in HPE research [8,9,10].

Deployed HPE is never a standalone network; in practice, it is one stage within a perception pipeline that begins at image acquisition—where sensor modality, device placement, frame rate, compression artifacts, and illumination jointly corrupt or constrain the input before a single forward pass occurs—and extends through person detection, pose inference, multi-person tracking, temporal smoothing, post-processing, hardware acceleration, and task-level decision logic [11,12,13]. Each stage introduces failure modes that propagate forward; calibration errors and insufficient temporal sampling can reduce the reliability of downstream pose estimates, particularly for fast-moving limbs and small joints that are important for action recognition and gait analysis.

Benchmark scores do not capture this: COCO AP averages over scale, occlusion, and crowd levels in ways that systematically favor clean, well-lit, mid-range captures—the conditions least representative of surveillance corridors, factory floors, or wrist-worn IMU-fused camera rigs. Markerless multi-view surveys and conventional 2D benchmark analyses both confirm that controlled evaluation settings cannot reproduce deployment variability [14,15]. This is not a calibration problem that better held-out splits can fix. Crowded scenes cause PAF-based bottom-up methods to misassemble skeletons, heavy occlusion drives top-down single-person regressors into anatomically implausible joint configurations, and low-resolution inputs collapse HRNet’s multi-scale fusion advantage entirely [16,17,18,19,20]. We contend that average metric scores actively conceal these failure modes rather than summarize them, and that any review treating benchmark performance as a proxy for operational reliability is analyzing a different problem than the one deployment engineers actually face.

Existing surveys examine HPE through task taxonomies, 2D and 3D estimation, multi-view or video settings, datasets, metrics, model architectures, and benchmark progress [1,14,21,22,23,24]. These contributions provide essential field-level coverage, but deployment is usually one topic among many rather than the organizing unit of analysis. This review therefore analyzes HPE as a deployable sensor-to-decision system: it identifies interacting data, system, task, and temporal gaps; examines the complete processing pipeline; compares method families under operational constraints; and links evaluation priorities to application-level risk. Section 2 states the structured narrative selection approach, and Table 1 positions this contribution relative to recent surveys.

The discussion is intended for both HPE researchers and practitioners who need to select or adapt pose estimation methods for real-world systems; it therefore avoids presenting recent architectures as a purely chronological progression, instead examining how different methodological choices affect latency, memory usage, temporal stability, and deployment reliability. This framing is particularly relevant for sensing and perception applications, where algorithmic advances are routinely evaluated alongside engineering feasibility, computational constraints, and application-specific utility.

A recurring observation throughout this review is that many HPE methods are developed under carefully controlled experimental conditions, whereas practical deployments must contend with imperfect imaging, limited hardware, domain-specific movement patterns, and long-term operational constraints. Accuracy, efficiency, and deployment reliability are therefore treated here as interconnected requirements rather than independent evaluation criteria.

This does not imply that benchmark accuracy is unimportant; rather, benchmark results should be interpreted in light of the conditions under which they were obtained and the computational costs required to reproduce them. For HPE to function reliably in applied systems, evaluation must connect model design, system integration, and domain-specific validation.

## 2. Review Scope and Literature Selection

### 2.1. Review Approach and Scope

This article is a deployment-oriented structured narrative review. It does not claim the exhaustive coverage, protocol registration, duplicate screening, or quantitative evidence synthesis expected of a systematic review or meta-analysis. Instead, representative literature is organized around an explicit analytical framework: the data, system, task, and temporal gaps that separate benchmark performance from operational reliability. The unit of analysis is therefore not only the pose-estimation network, but the complete sensor-to-decision pipeline and its application-level consequences.

### 2.2. Search Sources and Selection Principles

The literature base was assembled through relevance-oriented searches and reference tracing, followed by a supplementary structured search completed on 17 July 2026. Publicly accessible sources included Google Scholar, IEEE Xplore, the ACM Digital Library, and publisher platforms. Query families combined terms for human pose estimation with review or survey, deployment or real-world use, edge or mobile inference, latency, memory, power, temporal modeling or tracking, and the five application areas considered in this article (sports, rehabilitation, human–computer interaction, autonomous driving, and industrial safety). The main time window was 2014 to 17 July 2026; earlier foundational work was retained when required to explain an established method or metric.

Studies were included when they provided verifiable technical evidence directly relevant to HPE methods, deployment pipelines, device or system resources, temporal behavior, or application reliability. Primary research articles and authoritative benchmark papers were preferred for technical claims; recent reviews were used to position the contribution and identify coverage gaps. Records were excluded when they duplicated an already represented result, were only marginally related to HPE deployment, or lacked sufficient technical information to support the claim for which they were considered. Reference lists of included surveys and primary papers were traced to locate foundational or deployment-specific studies.

Evidence was synthesized qualitatively by mapping each source to the four deployment gaps, the seven-stage pipeline, model family, reported resource or latency information, temporal support, and application-level reliability requirement. Because input resolution, hardware, software backend, preprocessing, person count, precision mode, and timing boundary differ substantially across studies, runtime and resource values were not pooled as if they were directly comparable. Missing information is reported as N/R rather than inferred.

### 2.3. Positioning Relative to Recent Reviews

Recent surveys provide broad taxonomies of 2D and 3D HPE, benchmark datasets, evaluation metrics, multi-view or video methods, and application areas [1,14,21,22,23,24]. Table 1 clarifies the complementary focus of the present review. Its distinctive contribution is to connect model choice with the full sensing and deployment pipeline, diagnose failures through four interacting gaps, and translate that diagnosis into scenario-specific evaluation priorities and a minimum deployment reporting checklist.

The authors’ 2025 Applied Sciences review [24] is method-centered and limited to 2D HPE; it classifies architectures, summarizes datasets and evaluation metrics, and compares leading benchmark models. The present article does not reuse that review’s taxonomy or benchmark-comparison structure as its contribution. It instead addresses the end-to-end deployment problem across 2D, 3D, multi-person, and video settings, with explicit attention to sensing conditions, pipeline coupling, real-device constraints, temporal reliability, and downstream task risk. A text-overlap check was also performed during revision so that shared background concepts were independently expressed and the two articles remain substantively distinct.

This structured narrative approach has limitations. Relevance-oriented selection can introduce author judgment, and the review does not provide an exhaustive record count or a PRISMA-style screening flow. Publicly accessible, predominantly English-language literature may also underrepresent proprietary deployments and negative results. These boundaries are stated explicitly so that the framework is interpreted as a transparent deployment-oriented synthesis rather than a complete census of HPE research.

## 3. The Deployment Gap in Real-World Human Pose Estimation

### 3.1. Benchmark-to-Deployment Gap

The benchmark-to-deployment gap refers to the mismatch between controlled evaluation settings and real-world operational conditions. Standard HPE benchmarks typically provide curated annotations, fixed evaluation metrics, and relatively stable data distributions, which support fair comparison across methods. Deployed systems, however, must operate under sensor noise, domain shift, hardware limitations, temporal instability, and downstream task constraints that benchmarks do not capture. AP, PCK, AUC, and MPJPE are therefore necessary but insufficient indicators of deployment suitability [2,3,4,15].

**Figure 1:**
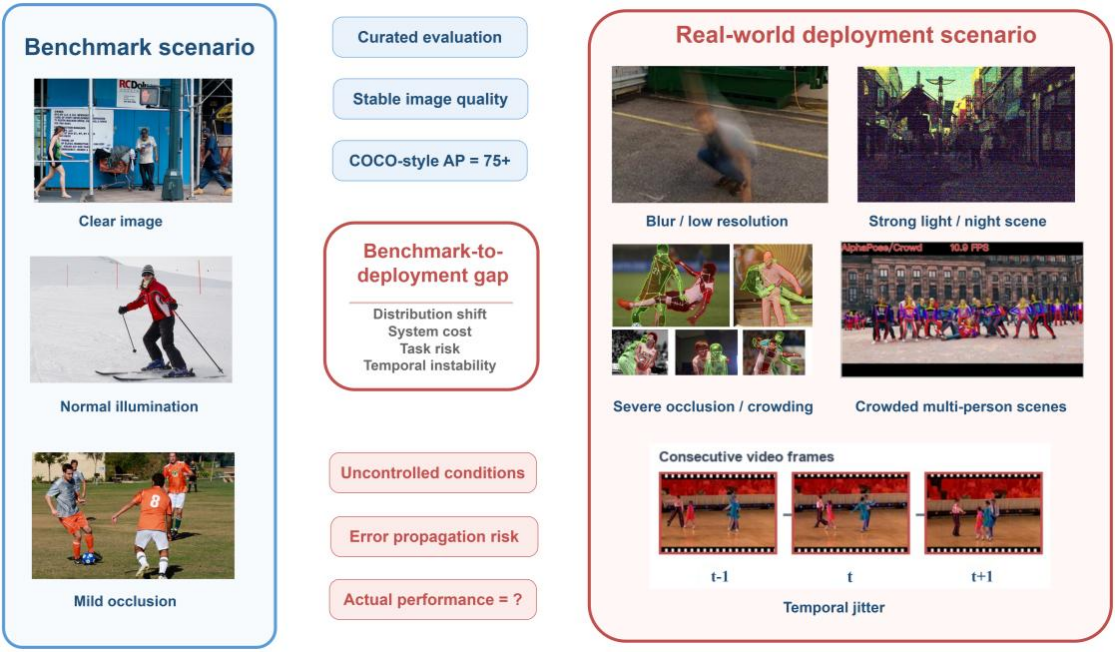
Comparison between benchmark scenarios and real-world deployment scenarios for human pose estimation.

This distinction is important because benchmark protocols are designed for reproducibility, whereas deployment environments are influenced by operational variability. In benchmark evaluation, images are collected, annotated, resized, and tested under a common protocol. Deployed systems must operate under continuously changing conditions, including camera placement, frame rate, compression quality, lighting, crowd density, and computational budget. A model may therefore achieve stable results in offline evaluation while producing temporally unstable predictions after integration into a real-time perception pipeline. Figure 1 illustrates the distinction between benchmark scenarios and real-world deployment scenarios under operational variability.

The benchmark-to-deployment gap should not be interpreted as a limitation of benchmarks themselves. Benchmarks remain essential because they enable standardized comparison and cumulative methodological progress. The main issue is that benchmark scores are often treated as direct indicators of real-world performance. A deployment-oriented review should therefore preserve the value of benchmark evaluation while identifying the practical conditions and system-level constraints that remain insufficiently represented in standard protocols.

This distinction can be framed in terms of controlled validity and operational validity: Controlled validity concerns whether a method performs well under a shared benchmark protocol. Operational validity concerns whether it remains stable when the visual environment, hardware backend, and downstream task conditions diverge from benchmark assumptions. HPE methods should be evaluated from both perspectives; the gap between them becomes particularly relevant in sensor-captured streams, where RGB-D modalities, depth sensing, camera placement, and mobile or wearable sensing conditions can alter the input distribution before the HPE model is applied [25,26].

#### 3.1.1. Data Gap

COCO, MPII, Human3.6M, and PoseTrack encode capture and annotation choices that may not represent operational conditions [2,3,4,15]. CrowdPose reports a higher average intersection over union (IoU) between human bounding boxes than MS COCO (0.27 versus 0.06), indicating substantially greater person overlap in CrowdPose [16]. Low-resolution and low-light evaluations likewise expose degradation that aggregate AP on standard images can conceal [18,19], while FreeMan expands outdoor 3D capture across more varied viewpoints and scales than controlled studio datasets [20]. Together, these studies show that crowding, resolution, illumination, viewpoint, and scale can affect both detection and pose inference; the magnitude and dominant failure stage depend on the pipeline and evaluation condition [16,17].

Aggregate accuracy does not fully characterize stability under deployment perturbations. Occlusion, resolution loss, illumination shift, viewpoint change, and crowding can interact through the pipeline rather than act as independent penalties: for example, degraded imagery may first reduce person-detection recall or shift the region of interest, after which the pose model receives missing or biased input. Image enhancement, multi-view acquisition, or stronger detectors may mitigate particular failures, but they also add computation, memory traffic, calibration requirements, or latency. Deployment claims should therefore specify the tested condition and pipeline boundary instead of extrapolating from a single benchmark score.

The data gap is application-specific in ways that a single difficulty axis cannot characterize. Sports footage can introduce motion blur around fast-moving wrists and ankles, precisely the joints used for stroke analysis and repetition counting. Home rehabilitation varies in camera placement, background, clothing, and lighting. Autonomous-driving footage combines long viewing distance, adverse weather, occlusion, and motion blur, while industrial monitoring adds helmets, reflective personal protective equipment, tools, elevated camera viewpoints, and equipment occlusion. These are structurally different conditions, not simply harder samples of one benchmark, and they change which joints and errors matter to the downstream task.

Sensor-side constraints make the data gap a hardware problem as much as a data problem. Sensor I/O bandwidth, on-device preprocessing latency, edge inference memory ceilings, and privacy-mandated local processing can each independently block the deployment of a model that achieves strong benchmark AP [13,27]. This intersection is almost never analyzed in benchmark papers, which report model accuracy in isolation from the acquisition and preprocessing pipeline that determines whether the model’s input ever resembles its training distribution.

Annotation semantics compound the deployment gap. COCO keypoints are evaluated with Object Keypoint Similarity (OKS), which normalizes localization error by object scale and keypoint-specific constants rather than applying a fixed pixel-distance rule [2]. That benchmark definition may still be misaligned with sports or rehabilitation tasks in which small systematic wrist, knee, or hip errors bias joint angles or repetition counts. Similarly, mean per-joint position error can conceal joint-specific bias that matters to gait assessment, and generic AP does not directly encode the downstream consequence of a missed or uncertain pedestrian cue. Domain-aware validation should therefore state which joints, visibility conditions, temporal properties, and task outputs are evaluated.

#### 3.1.2. System Gap

HPE does not deploy as a network—it deploys as a pipeline, and this distinction is where most benchmark-to-deployment gaps originate. A production system integrates sensor acquisition, person detection, bounding-box localization, pose inference, multi-person tracking, temporal smoothing, coordinate decoding, and application-level post-processing into a sequential chain, where each stage’s failure mode is the next stage’s corrupted input. RTMPose, BlazePose, and EfficientPose each demonstrate that acquisition, detection, and inference must be co-optimized rather than benchmarked in isolation [8,28,29]. OpenPose, SORT, DeepSORT, and PoseFlow make the downstream consequence explicit: missed detections, inaccurate crops, and identity switches propagate into temporal inconsistency and degraded downstream reliability at rates that per-frame pose AP cannot predict [30,31,32,33].

Multi-person and real-time settings expose the system gap sharply. In top-down pipelines, the cost of pose inference usually grows with the number of detected people, although the practical rate depends on batching, input size, hardware, and backend. Bottom-up and one-stage approaches avoid repeated per-person inference but can introduce keypoint-grouping and association errors as overlap increases [34,35,36]. SORT, DeepSORT, and PoseFlow further show that useful temporal output depends on association and identity consistency as well as frame-level pose accuracy [31,32,33]. A high-accuracy per-frame estimate can still yield unusable motion trajectories when detector misses, fragmented tracks, or identity switches are frequent.

FLOPs and parameter counts are useful descriptors, but they do not by themselves determine real-device latency. Operator support, memory access, input resolution, precision mode, batching, preprocessing, post-processing, and software–hardware mapping can reverse the ranking suggested by theoretical complexity. CNNs often benefit from mature kernels and deployment toolchains, yet that advantage is conditional on the target backend and does not guarantee lower latency for a complete pipeline. For deployment studies, the most informative runtime measurement is end-to-end latency with an explicit boundary that includes the stages used in the application. Where such data are unavailable or incomparable, they should be reported as not reported rather than reconstructed from backbone timing.

Error propagation and recovery are system properties that frame-level pose metrics do not isolate. In an operational pipeline, a detector miss can fragment a track, create an identity switch, and bias downstream motion statistics after visually plausible keypoints reappear. The duration and severity of this effect depend on tracker logic, association thresholds, occlusion length, and re-initialization policy. Deployment-oriented evaluation should therefore measure recovery after controlled failures and state the definition of recovery, instead of assuming that one incorrect frame has only a one-frame consequence.

#### 3.1.3. Task Gap

The task gap refers to the mismatch between benchmark objectives and application requirements. Standard evaluation focuses on keypoint localization accuracy or 3D reconstruction error, but healthcare, sports, and rehabilitation systems may require range-of-motion measurement, gait analysis, or action quality assessment [37,38,39]. Transportation applications use pose information for pedestrian intention estimation and risk interpretation [40,41,42,43]. Safety-oriented systems may need to detect falls, monitor worker behavior, or verify PPE compliance [44,45]. Across these domains, the consequence of a localization error depends on which joint is affected, at what phase of movement, and within what decision context. Average keypoint distance metrics do not capture these factors.

Deployment-oriented evaluation should therefore include task-level reliability assessment. Small localization errors in the knee or wrist may have limited influence on aggregate AP but can alter rehabilitation measurements, sports technique evaluation, gesture commands, or industrial safety alerts. The same HPE model may therefore be acceptable for offline video annotation while remaining unsuitable for clinical monitoring, real-time interaction, or safety-critical perception systems.

In sensor-based video streams, temporal instability may also result from frame-rate variation, dropped frames, motion blur, rolling-shutter artifacts, and synchronization mismatches between acquisition and inference modules [25,46].

The task gap is also related to interpretability. In many practical applications, users require not only pose outputs but also estimates of whether the predictions are reliable enough to support downstream decisions. For example, clinicians may require confidence in joint-angle measurements, coaches may require stable phase segmentation across repeated motions, and autonomous systems may need to determine whether pedestrian pose cues remain reliable under occlusion. These requirements are rarely reflected in conventional benchmark metrics.

Task-level mismatch can also change the relative importance of different joints. General pose benchmarks usually evaluate all annotated keypoints under a common protocol, whereas practical applications may depend heavily on a small subset of joints or on derived biomechanical quantities. In upper-limb rehabilitation, shoulder and elbow angles may dominate clinical interpretation. In pedestrian intention estimation, body orientation and limb configuration may be more relevant than precise ankle localization. These observations suggest that deployment-oriented evaluation should incorporate weighted, task-specific, or downstream-oriented metrics when appropriate.

#### 3.1.4. Temporal Gap

Frame-level evaluation alone is insufficient for systems that operate on continuous video. PoseTrack, VideoPose3D, FAMI-Pose, and temporal difference learning motivate sequence-level properties such as trajectory stability, occlusion recovery, identity consistency, and downstream signal jitter [4,47,48,49]. A model can achieve strong frame-level accuracy while producing trajectories that are unsuitable for angular velocity, gait, or action analysis. The relevant evaluation window and metric should match the operational duration and task; current studies do not use a single standardized protocol for long-duration drift or recovery.

Temporal modeling can reduce jitter and improve motion continuity, but its deployment cost depends on causality, buffering, window design, update schedule, and hardware. A causal model may begin producing outputs after warm-up and then update every frame, whereas a centered or non-causal window requires future frames and adds unavoidable look-ahead latency. Longer sequences also increase memory and computation for many attention-based designs, although the exact cost is implementation-dependent. Accordingly, temporal accuracy should be reported together with algorithmic look-ahead, buffering, end-to-end response latency, and the hardware/backend used.

The failure modes differ by mechanism, and no temporal method is universally preferable. Low-pass filters trade jitter reduction against lag and attenuation of rapid motion; their effective behavior depends on sampling rate, motion spectrum, cutoff choice, and whether parameters adapt to signal speed. The 1€ filter, for example, explicitly adapts the cutoff to balance jitter and lag [50]. HPE papers rarely report a common filter bandwidth or motion-frequency failure threshold, so such a threshold should not be inferred across methods. Sequence models can support short-term occlusion recovery but depend on temporal receptive field and causality, while trackers can fail through fragmentation or identity switches as scene ambiguity increases [31,32,33]. Comparisons should therefore specify motion regime, filter or window settings, latency, and failure conditions.

Extending temporal context does not resolve the temporal gap—it relocates it. The appropriate temporal design is determined entirely by operational mode: offline analysis systems can exploit full future context and arbitrarily large windows without latency penalty, while interactive and safety-critical systems must deliver stable outputs within a fixed response budget that is defined by the application, not by the model designer. This distinction is rarely stated explicitly in temporal HPE papers, which report sequence-level accuracy without specifying whether the results assume causal inference, fixed-latency buffering, or offline post-processing. Comparing VideoPose3D’s non-causal dilated convolutions against an online smoothing filter without declaring the operational mode is not a controlled comparison—it is an ambiguous one, and the field’s current reporting norms routinely permit it.

**Figure 2:**
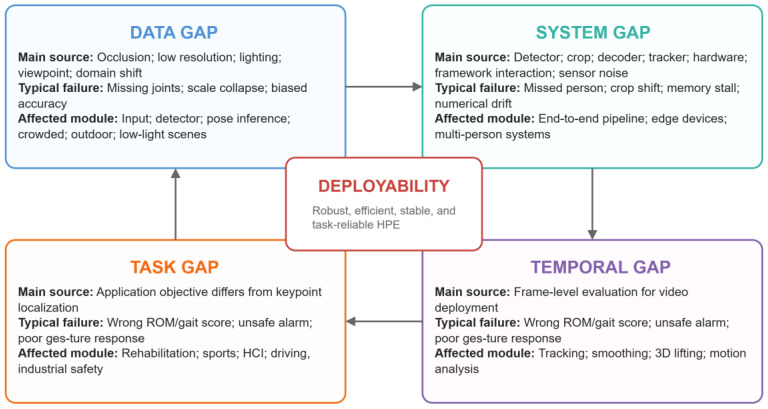
Deployment-oriented four-gap framework for real-world HPE systems.

The four gaps should not be treated as isolated problems; Figure 2 summarizes their interactions and illustrates how they jointly affect real-world HPE deployment.

### 3.2. Framework of Deployment Gaps

The four gaps are analytically separable but operationally entangled. The data gap reflects visual and distributional mismatch, the system gap arises from pipeline integration and hardware constraints, the task gap concerns application-level objective misalignment, and the temporal gap relates to sequence stability and trajectory coherence; treating them as independent failure modes produces misdiagnosis. Low-light conditions appear initially as a data problem, but compensating through image enhancement increases the computational cost, raises end-to-end latency, and may still fail to meet task-level reliability thresholds—a single environmental condition simultaneously activating three of the four gaps simultaneously.

The four-gap framework functions as a diagnostic instrument, not a taxonomy. When a deployed HPE system underperforms, the root cause is rarely self-evident: distributional mismatch, detector bottleneck, metric-objective misalignment, and video-output instability produce overlapping symptoms but require categorically different interventions. Diverse training data address the data gap and do nothing for a detector recall ceiling. INT8 quantization reduces inference latency but cannot correct a pose metric that is structurally misaligned with the downstream clinical or safety objective. Temporal smoothing suppresses per-frame jitter but introduces response delay that interactive and safety-critical systems cannot absorb, and applying it to a system whose primary failure is distributional mismatch wastes the engineering effort entirely.

The framework also prevents the overgeneralization that single-axis method comparisons routinely produce. A model improving occlusion robustness by 6 AP on OCHuman is operationally useless for wearable deployment if its memory footprint exceeds the target device’s DRAM budget. Lightweight architectures—e.g., Lite-HRNet and MobileNet-based pose models—ease system-level integration but fail in clinical rehabilitation contexts where hip and knee localization precision drives range-of-motion measurement validity. Temporal models stabilize video output at the cost of latency that disqualifies them from real-time HCI or pedestrian-risk pipelines. We contend that none of these trade-offs are visible in benchmark comparisons that report a single accuracy metric without specifying the deployment context—which describes the majority of published HPE evaluations.

The four-gap framework does not replace conventional method taxonomy—it recontextualizes it. CNN-based methods carry lower system-level integration risk because they depend on mature operators and optimized inference libraries with broad hardware backend support. Transformer-based architectures—e.g., ViTPose and PoseFormer—address structural ambiguity in complex multi-person scenes but increase computational and memory costs in ways that frequently exceed edge deployment budgets. Temporal models suppress prediction jitter and improve trajectory coherence, but their latency overhead and non-causal variants’ dependence on future frames make them incompatible with strict online constraints. Lightweight models improve edge feasibility directly, but their keypoint accuracy under domain shift requires explicit validation rather than the assumption that COCO AP transfers to the target operating condition. Each method family addresses a subset of the four gaps while exacerbating others, and recognizing this structure is the prerequisite for making deployment decisions that hold in practice.

## 4. Real-World Human Pose Estimation System Pipeline

### 4.1. System Overview: Real-World HPE Is Not a Single-Module Task

A real-world HPE system is an end-to-end perception pipeline, not an isolated pose estimator. A typical pipeline begins with camera or video input and proceeds through human detection or region localization, pose inference, temporal association, smoothing, coordinate transformation, and task-specific interpretation. Because each stage feeds directly into the next, errors propagate and accumulate. A model with strong single-frame benchmark accuracy can still fail in deployment if the detector misses a person, the crop is misaligned, the tracker switches identities, or post-processing introduces excessive latency.

This pipeline perspective also changes the evaluation target. Instead of focusing only on which pose backbone achieves the highest AP or lowest MPJPE, deployment-oriented HPE evaluates whether the complete system can satisfy application-specific constraints on latency, stability, memory usage, power consumption, and operational reliability. The following subsections summarize the major pipeline modules and deployment bottlenecks.

The pipeline structure also determines where optimization is most necessary. In some systems, the pose network dominates runtime cost, whereas in others, detection, cropping, video decoding, tracking, or post-processing consumes a comparable fraction of the execution time. A deployment-oriented design should therefore profile the complete pipeline before selecting a model family. This is particularly important in multi-camera systems, crowded scenes, and mobile or embedded platforms, where memory bandwidth and operator availability may become more restrictive than raw computational throughput.

For camera-based and edge sensing systems, profiling should also account for sensor bandwidth, acquisition frame rate, preprocessing latency, and the location of inference execution, including on-device, edge-side, or cloud-based deployment [13,27].

Another implication of the pipeline perspective is that module-level improvements should be evaluated within the context of the complete system. Replacing a pose backbone with a more accurate model may provide limited practical benefit if detection errors remain the dominant failure source. Similarly, introducing a stronger tracker may not improve downstream application performance when coordinate noise from the pose estimator dominates the measurement process. System design should therefore identify the primary bottleneck before introducing additional model complexity.

The pipeline perspective also highlights a distinction between algorithmic modularity and deployment modularity. A system may be modular at the implementation level, yet its errors remain tightly coupled in practice. A bounding-box shift alters the input distribution of the pose estimator, pose estimation errors propagate into tracker states, and tracking failures distort downstream temporal statistics. This interdependence is precisely why real-world HPE should be evaluated as an integrated system rather than as a collection of independent modules.

**Figure 3:**
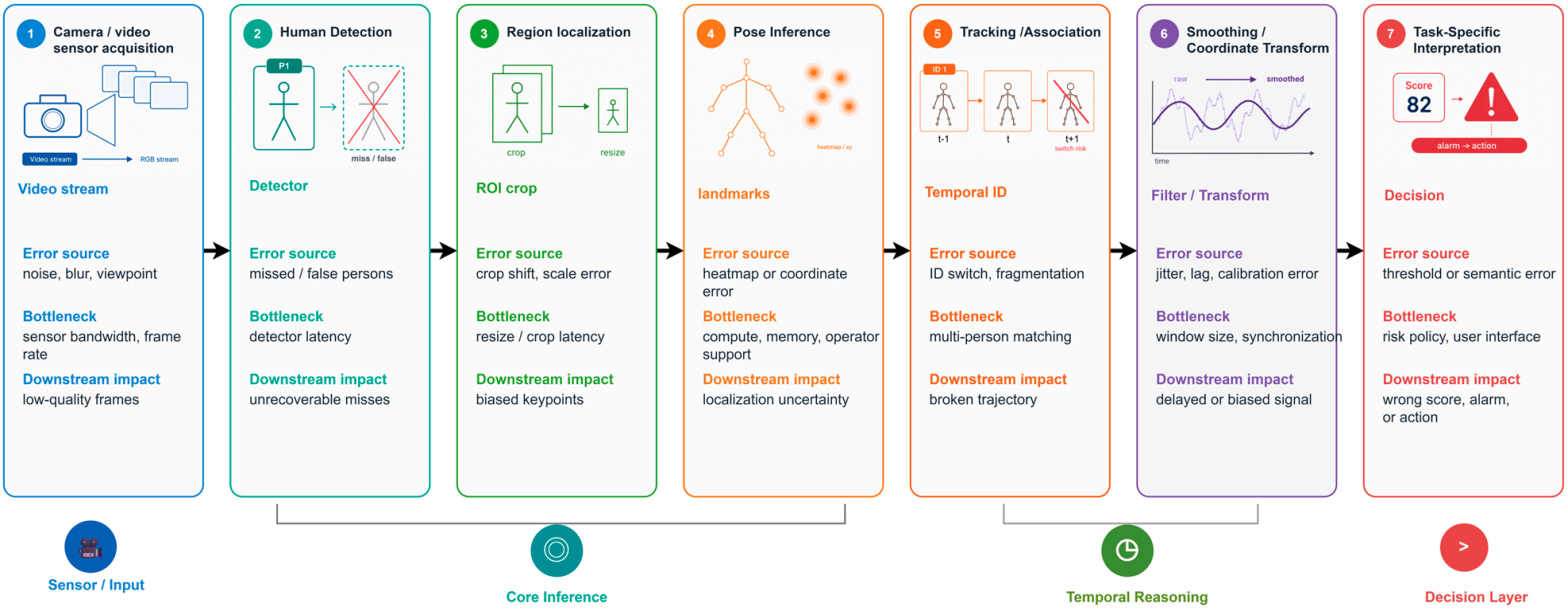
Errors, bottlenecks, and downstream effects may propagate across interconnected stages during deployment.

Deployed HPE systems operate as multi-stage sensor-to-decision perception pipelines. Figure 3 summarizes the complete pipeline and identifies where errors, bottlenecks, and downstream effects tend to emerge.

### 4.2. Human Detection and Localization

In top-down HPE, human detection serves as the entry point for pose inference. Each person must be localized before single-person pose estimation can proceed, which provides strong accuracy and flexibility but scales poorly with the number of detected people. Detection failures, inaccurate bounding boxes, and repeated cropping or resizing can directly degrade downstream keypoint localization [5,34].

Bottom-up and one-stage approaches reduce reliance on repeated per-person inference by predicting keypoints or multi-person poses more directly [35,36]. This makes them attractive in crowded scenes and real-time systems, but they introduce their own challenges in grouping, parsing, and representation, particularly under occlusion and scale variation. The detection and localization stage should therefore be evaluated together with the pose module rather than treated as a preprocessing step.

Region localization also affects numerical stability. Bounding-box expansion, aspect-ratio normalization, image resizing, and coordinate transformation collectively determine how image-space information is mapped into the pose estimator and projected back to the original frame. Small inconsistencies in these steps may be invisible during qualitative inspection but can influence joint-angle estimation, action scoring, and frame-to-frame smoothness. Deployment-oriented studies should therefore report not only the pose backbone but also the complete preprocessing and coordinate-decoding pipeline.

Localization design also has implications for system scalability in multi-person scenes. In sparse scenes, top-down systems can produce accurate and interpretable results because each person is processed independently. In crowded scenes, repeated inference, overlapping bounding boxes, and detector uncertainty become the dominant computational burden. Bottom-up and one-stage methods are more suitable under these conditions, provided that keypoints can still be grouped or represented reliably under occlusion. The appropriate choice depends on scene characteristics and the specific constraints of the deployment setting.

### 4.3. Pose Inference and Decoding

The pose estimation module is the central inference component of the pipeline; it converts cropped human regions or image-level features into 2D keypoints, 3D joints, heatmaps, coordinate distributions, or temporal pose sequences. CNN-based methods such as SimpleBaseline, HRNet, DeepPose, CPN, and HigherHRNet demonstrate that localization accuracy depends heavily on backbone design and high-resolution feature representations [5,6,51,52,53]; methods including DARK, UDP, DEKR, SimCC, and RLE further show that heatmap encoding, coordinate decoding, and data-processing consistency can affect prediction stability and deployment behavior [54,55,56,57,58].

Transformer-based and real-time systems expand the design space in different directions. ViTPose, RTMPose, RTMO, ViT, and Swin Transformer reflect varying trade-offs among model scale, global representation capability, and deployment cost [7,8,36,59,60]. TransPose, TokenPose, PRTR, HRFormer, and PPT examine how keypoint tokens, high-resolution attention, and token pruning affect estimation efficiency and computational overhead [61,62,63,64,65]. In deployment scenarios, these methods should be assessed by their contribution to end-to-end system performance rather than backbone accuracy alone.

The inference module is also where the tension between accuracy and efficiency is most direct. High-resolution feature maps improve localization accuracy but increase memory traffic. Heatmap-based representations yield spatially interpretable predictions but require additional decoding steps. Direct coordinate regression or classification-based representations can reduce post-processing overhead, though they may introduce different error patterns. These design choices should be evaluated under the constraints of the target deployment scenario rather than being selected from benchmark rankings alone.

Training and calibration strategies interact closely with this module as well. Data augmentation, input resolution, flip testing, test-time refinement, and confidence calibration can all influence deployment behavior. Some techniques improve benchmark scores but increase latency or create discrepancies between offline evaluation and actual runtime operation. A deployment-oriented system should therefore distinguish between the accuracy obtained under the full evaluation pipeline and what is achievable under the runtime configuration used in practice.

### 4.4. Tracking and Temporal Smoothing

Video-based HPE requires temporal consistency across consecutive frames. Tracking and smoothing modules are commonly used to associate identities, reduce keypoint jitter, recover from short-term occlusion, and produce stable trajectories for downstream analysis. PoseTrack, Detect-and-Track, and VideoPose3D illustrate the importance of sequence-level tracking and temporal lifting for achieving these properties [4,47,66]. Transformer-based temporal methods such as PoseFormer, MHFormer, and MixSTE show that temporal modeling can improve 3D pose representations [67,68,69]. Later methods, including STCFormer, PoseFormerV2, and MotionBERT, further refine spatiotemporal and motion representations [70,71,72].

Temporal processing also changes the system latency profile. Causal smoothing can operate online but provides limited long-range temporal context. Bidirectional or long-window models improve temporal stability but require future frames or larger frame buffers. SSM/Mamba-based models, including S4, Mamba, and PoseMamba, provide a potential direction for long-sequence modeling in HPE [10,73,74]. Vision-oriented variants such as Vision Mamba, VMamba, LocalMamba, and VideoMamba further suggest broader potential for visual and video sequence modeling; their practical value for real-device HPE deployment, however, still requires systematic evaluation and validation [75,76,77,78].

In deployment scenarios, temporal modules should be selected according to the required response mode. Offline sports analysis or clinical review can benefit from bidirectional temporal models because future frames are available during inference. Live interaction, mobile feedback, and safety monitoring typically require causal processing, bounded buffering, and predictable latency. In these contexts, the distinction between online and offline temporal modeling is often more consequential than the absolute accuracy gain that a temporal model reports on an offline benchmark.

Temporal association also introduces a class of errors that frame-level evaluation does not capture. A system may estimate keypoints accurately within individual frames but assign them to the wrong person following occlusions or trajectory crossings. Such identity switches can significantly affect gait analysis, worker monitoring, and pedestrian behavior understanding. Video HPE systems should therefore report identity consistency and recovery behavior in addition to frame-level keypoint accuracy.

### 4.5. System Bottlenecks and Design Principles

The major bottlenecks of real-world HPE are distributed throughout the pipeline. Detection can introduce missed subjects and scale estimation errors, and pose inference can introduce localization and decoding errors. Temporal association can lead to identity switches and smoothing delays, while compression and acceleration may introduce numerical drift. Application modules can further amplify small keypoint errors into task-level failures. Lightweight architectures such as Lite-HRNet, MobileNets, ShuffleNet, and LitePose are therefore important for deployment under resource constraints [9,79,80,81]. Quantization and deployment frameworks, including ONNX Runtime, TensorRT, and Core ML Tools, should also be evaluated on target hardware rather than assessed only through FLOPs or parameter counts [82,83,84,85,86].

A deployment-oriented HPE study should define its sensor input and timing boundary; identify every enabled pipeline stage; report the target hardware, framework, backend, precision, and batch size; and measure end-to-end latency, throughput, memory, and power where relevant. It should also report robustness conditions, temporal stability, recovery behavior, and task-level outputs. Together, these elements constitute a minimum reporting checklist.

These principles suggest a different approach to method comparison. Instead of ranking models using a single metric, researchers should describe the operating conditions under which a method is expected to perform reliably, such as sparse or crowded scenes, static images or video streams, offline or online inference, cloud or edge hardware, and visualization or decision-support tasks. Such reporting would make HPE studies more useful for engineers and domain users who must select methods under concrete deployment constraints.

Practical deployment requires evaluation beyond model-level accuracy. The complete pipeline should be tested end-to-end on the target hardware and under the visual conditions expected in the application domain. Evaluation should also include application-level outputs, such as joint-angle measurements, gesture decisions, pedestrian-risk estimation, or safety-event detection. These criteria help relate algorithm selection to the operational value of the deployed system. Table 2 summarizes the major pipeline modules, bottlenecks, failure propagation patterns, evaluation metrics, and mitigation strategies in real-world HPE systems.

## 5. Methods for Deployment-Oriented Human Pose Estimation

### 5.1. CNN-Based Methods

CNN-based methods have long been the mainstream approach for human pose estimation, and their continued presence in real-world systems reflects not only strong benchmark performance but also a relatively mature engineering ecosystem and deployment stability. Early DeepPose formulated HPE as a direct regression task from input images to joint coordinates, demonstrating that deep neural networks could predict pose in an end-to-end manner [51]. Later methods, including convolutional pose machines and stacked hourglass networks, introduced multi-stage refinement, heatmap regression, and cross-scale feature integration, gradually shifting HPE from coordinate regression toward heatmap-based localization [87,88]. For deployment-oriented systems, heatmap regression offers a more interpretable spatial representation of keypoint probability distributions and tends to produce more stable optimization during training; it is also generally more tolerant to local spatial ambiguity than direct coordinate regression. These advantages, however, come at the cost of maintaining sufficient spatial resolution and the additional computation associated with upsampling, coordinate decoding, and post-processing.

CNN-based multi-person pose estimation has evolved into two main engineering paradigms: top-down and bottom-up. OpenPose uses part affinity fields to associate detected keypoints with body limbs across multiple people, making it a representative bottom-up framework for real-time multi-person pose estimation [30]. Associative embedding integrates keypoint detection and instance grouping within a single detection-and-grouping framework [35]. RMPE, CPN, SimpleBaseline, and related methods follow the top-down paradigm, which first detects human instances and then applies single-person pose estimation within each bounding box [5,34,52]. Bottom-up methods avoid repeatedly executing a single-person pose network as the number of people grows, which makes them more suitable for real-time inference in crowded scenes; their grouping and post-processing stages, however, can become computational bottlenecks under heavy occlusion, dense crowds, or small-scale instances. Top-down methods generally provide higher localization accuracy and a cleaner modular separation between detection and pose estimation, but overall latency depends on both detector performance and the number of detected people.

Within high-precision CNN-based architectures, HRNet improves keypoint localization by maintaining high-resolution feature representations throughout the network and repeatedly fusing multi-scale features [6]. HigherHRNet extends this design to bottom-up multi-person pose estimation and mitigates scale variation through high-resolution feature pyramids, with particular benefits for small-scale human localization [53]. DARK and UDP further show that deployment performance in HPE is influenced not only by the backbone architecture but also by implementation details such as heatmap encoding, coordinate transformation, flip testing, and coordinate decoding [54,55]. These studies highlight an important deployment issue: small keypoint coordinate offsets in engineering systems may propagate to downstream tasks such as action scoring, rehabilitation assessment, and human–computer interaction. As a result, HPE accuracy in real-world deployment depends not only on backbone representation capability but also on the consistency of data processing and post-processing pipelines.

CNN-based methods also introduce several deployment trade-offs. High-precision architectures often rely on large input resolutions or high-resolution feature maps, which increase memory consumption and bandwidth requirements. The local receptive field of convolutions limits their ability to capture complex occlusion patterns, multi-person interactions, and long-range structural dependencies between body joints. Heatmap-based methods generally provide accurate keypoint localization, but their high-resolution outputs, upsampling operations, and coordinate decoding stages add inference latency and post-processing overhead. Methods such as DEKR, SimCC, and RLE redesign pose representations through dense regression, coordinate classification, or probabilistic regression to reduce quantization error, simplify post-processing, and address the efficiency bottlenecks associated with heatmap-based pipelines [56,57,58].

CNN-based methods remain a practical choice for deployment primarily because of their engineering maturity. Optimized convolution operators, broad hardware support, and well-established toolchains for quantization and inference acceleration make them suitable for edge devices, industrial vision systems, real-time interaction, and applications with strict stability requirements. Their main limitations are weaker global dependency modeling, limited cross-domain generalization, and the computational and memory overhead of high-precision variants. In this sense, their deployment advantages stem more from hardware friendliness, implementation stability, and engineering maintainability than from modeling capacity under all complex conditions. Table 3 summarizes representative HPE methods from a deployment perspective, covering deployment advantages, cost drivers, limitations, optimization leverage, and suitable application scenarios.

### 5.2. Transformer-Based Methods

Transformer-based methods were introduced into human pose estimation to address the limited capability of CNNs in modeling long-range dependencies. Human pose estimation relies not only on local texture cues but also on structural relationships among body joints, including the head, shoulders, elbows, wrists, hips, knees, and ankles. ViT demonstrated that image patches can be treated as sequential inputs for Transformer architectures, while Swin Transformer reduced the computational cost of high-resolution vision tasks through shifted-window attention [59,60]. Building upon these advances, HPE methods such as TransPose, TokenPose, PRTR, HRFormer, and ViTPose incorporated Transformer architectures or token-based representations into keypoint localization, extending structural relationship modeling beyond the local receptive fields of CNNs [7,61,62,63,64].

Beyond improving representation capability, Transformer-based methods have also attracted attention in deployment-oriented HPE systems because of their scalability and ability to model global structural relationships. TokenPose explicitly represents each keypoint as a token, allowing the model to jointly learn appearance features and inter-joint constraints within a unified representation [62]. PRTR formulates pose estimation as a cascaded Transformer regression problem, reducing dependence on manually designed post-processing stages [63]. HRFormer combines high-resolution representations with local window attention to improve computational efficiency while preserving spatial detail [64]. ViTPose demonstrates that a plain ViT backbone can achieve strong scalability and transferability in HPE, providing a practical baseline for high-accuracy pose estimation [7]. Despite their strong representation capability, these methods generally require substantial computational resources, making them more suitable for cloud servers, high-end GPUs, offline analysis pipelines, and scenarios where accuracy takes priority over latency and computational cost.

However, Transformer-based methods also introduce substantial deployment overhead. The memory and computational complexity of self-attention increase rapidly with the number of tokens, particularly for high-resolution inputs. Large Transformer models often depend on large-scale pretraining; they also require computationally intensive training configurations, increasing reproduction and transfer costs across datasets and deployment environments. In addition, operators such as attention, LayerNorm, and GELU remain less optimized than convolution on some edge hardware platforms, making quantization and low-precision inference more difficult. PPT reduces unnecessary attention computation on background regions through token pruning, suggesting that redundant global attention remains a major deployment bottleneck in Transformer-based HPE [65].

As a result, recent deployment-oriented HPE research has increasingly shifted from simply scaling Transformer architectures toward improving end-to-end system efficiency. Methods such as RTMPose and RTMO should therefore be viewed not as conventional “pure Transformer” approaches but, rather, as engineering-oriented HPE systems designed around real-time deployment constraints. RTMPose jointly optimizes model architecture, training strategy, deployment configuration, and inference efficiency, and it reports real-time performance across CPU, GPU, and mobile devices [8]. RTMO addresses the increasing inference cost of top-down multi-person pose estimation as the number of subjects grows, proposing a one-stage real-time framework that seeks a more practical balance between accuracy and inference speed for deployment scenarios [36]. These methods indicate that recent progress in HPE deployment depends not only on stronger backbones but also on coordinated optimization across model architecture, training strategy, output representation, and inference systems.

Overall, Transformer-based HPE methods provide strong global representation capability and achieve high accuracy in complex pose estimation tasks; however, their deployment on edge devices, low-power platforms, and large-scale real-time video systems remains challenging because of attention complexity, memory consumption, quantization difficulty, and dependence on large-scale pretraining. Consequently, practical deployment increasingly relies on additional optimization strategies, including lightweight architecture design, token pruning, knowledge distillation, low-bit quantization, and hardware-specific inference acceleration. Table 4 summarizes the deployment-oriented characteristics of representative Transformer-based HPE methods.

### 5.3. Temporal Modeling Methods

Real-world human pose estimation is typically deployed in continuous video streams rather than in independent static images. As a result, high single-frame AP does not necessarily translate into stable video-level predictions. Common deployment issues include keypoint jitter, failure to recover after short-term occlusion, incorrect person association, discontinuous motion trajectories, and instability in downstream motion analysis. PoseTrack is a large-scale benchmark for video pose estimation and human pose tracking; it emphasizes cross-frame trajectory consistency and temporal keypoint stability, highlighting that real-world HPE should be evaluated beyond frame-level accuracy and toward sequence-level stability [4].

Early video HPE methods typically adopted a detect–estimate–associate pipeline. Detect-and-track methods estimate keypoints on individual frames or short clips and generate cross-frame pose trajectories through lightweight tracking [66]. These pipelines integrate naturally with detectors, trackers, and post-processing modules, making them straightforward to deploy in practice; their main limitation is error propagation across the detection, pose estimation, and tracking stages. System latency is also governed by the full pipeline rather than the pose estimation network alone. In multi-person video scenarios, stable temporal association is as important as per-frame pose accuracy.

For 3D HPE and 2D-to-3D pose lifting, TCN-based methods are generally more suitable for deployment-oriented temporal modeling. VideoPose3D uses dilated temporal convolutions to estimate 3D poses from 2D keypoint sequences, showing that such models can capture temporal dependencies at relatively low computational cost [47]. TCN-based models offer several practical advantages, including regular network structures, efficient parallel computation, and predictable inference latency, making them well suited for online or near-real-time systems; their main limitation is that the temporal receptive field is bounded by the input window length and dilation strategy, constraining their ability to handle long-duration actions, complex periodic motion, and long-term occlusion recovery.

Temporal Transformer-based methods further improve the modeling of long-range spatiotemporal dependencies. PoseFormer integrates spatial joint relationships and temporal dependencies within a Transformer framework [67]. MHFormer introduces a multi-hypothesis mechanism to alleviate depth ambiguity in monocular 3D HPE [68], while MixSTE alternately models spatial correlations and temporal motion patterns [69]. STCFormer reduces the computational cost of global spatiotemporal modeling through spatiotemporal criss-cross attention [70]. PoseFormerV2 improves efficiency by compressing long-sequence representations in the frequency domain [71], and MotionBERT extends HPE toward more general human motion understanding through large-scale motion representation pretraining [72]. These models improve temporal stability and representation capacity in video HPE, but they also increase GPU memory consumption, caching overhead, and inference latency.

Temporal Transformer-based methods are generally more suitable for cloud-based video analytics, motion assessment, rehabilitation analysis, and offline 3D pose reconstruction than for strict real-time edge deployment scenarios. Models that depend on future frames are difficult to integrate into causal online systems. Long temporal windows also increase response latency, while complex spatiotemporal attention applied to each frame introduces additional memory consumption and throughput constraints. Temporal modeling can improve stability and motion consistency in real-world HPE systems, but it also introduces additional deployment costs and system-level bottlenecks.

SSM- and Mamba-based approaches have attracted attention because they attempt to balance long-sequence modeling capability with computational efficiency. S4 investigates long-range sequence modeling through structured state-space models [73], while Mamba introduces selective state spaces and emphasizes linear sequence scaling together with hardware-aware implementation [74]. In computer vision, Vision Mamba, VMamba, LocalMamba, and VideoMamba extend Mamba/SSM architectures to visual tasks through bidirectional scanning, two-dimensional selective scanning, window-based scanning, and video representation modeling, respectively [75,76,77,78]. These studies provide a potential foundation for long-sequence video HPE, but they should not be regarded as mature deployment solutions for real-world HPE systems.

PoseMamba applies pure SSM-based architectures to monocular video 3D human pose estimation and explores long-sequence pose modeling through a bidirectional global–local spatiotemporal state-space module [10]. This indicates that Mamba/SSM-based approaches have started to enter the HPE field, although the current evidence remains substantially more limited than that available for CNN-, ViT-, and mature temporal Transformer-based approaches. Linear computational complexity also does not necessarily translate into faster real-world deployment. Actual inference speed still depends on operator implementation, inference frameworks, hardware backends, batch size, sequence length, and the complete end-to-end pipeline. Mamba/SSM should therefore be regarded as a potential direction for long-sequence video HPE, rather than as a fully validated deployment alternative. Table 5 summarizes the deployment-oriented trade-offs of representative temporal modeling methods in HPE, including their computational characteristics, latency patterns, deployment advantages, optimization potential, and suitable application scenarios.

### 5.4. Model Compression and Acceleration

Lightweight optimization is a practical engineering strategy for deploying high-precision HPE models in real-world systems. Instead of introducing entirely new pose estimation paradigms, this line of work reduces deployment cost through lightweight architectures, knowledge distillation, pruning, quantization, and inference optimization, allowing previously expensive models to run on mobile devices, edge platforms, and real-time systems. MobileNets and ShuffleNet introduced several core design principles for lightweight CNNs, including depthwise separable convolution, pointwise group convolution, and channel shuffle [79,80]. These lightweight vision designs provide the basis for HPE model compression, although HPE remains more sensitive to spatial resolution and local numerical errors because keypoint localization depends heavily on fine-grained spatial information.

Lite-HRNet and LitePose are representative lightweight architectures designed specifically for HPE. Lite-HRNet combines high-resolution representations with efficient shuffle blocks to reduce computational cost while preserving keypoint localization accuracy [9]. LitePose focuses on real-time multi-person pose estimation on edge devices; it identifies redundancy in the high-resolution multi-branch structure of HRNet under low-computation settings and replaces it with a more efficient single-branch architecture to improve edge-side inference latency [81]. The main advantage of these methods is that computational reduction is incorporated directly into the model design stage, rather than being applied through post-training compression.

Knowledge distillation provides another approach for transferring the capability of high-precision HPE models to lightweight models. OKDHP applies online knowledge distillation within a single-stage multi-branch network to transfer human pose structure knowledge, reducing part of the overhead associated with the conventional teacher–student two-stage pipeline [89]. Unlike image classification, HPE distillation must preserve not only the final prediction output but also heatmap distributions, joint structure relationships, intermediate features, and human topology information. For deployment, the main advantage of distillation is that small models can inherit spatial structure knowledge from larger models while maintaining acceptable pose estimation accuracy under lower computational budgets.

Token pruning plays an important role in reducing redundant computation in Transformer-based HPE. PPT addresses background token overhead by identifying human body regions and restricting self-attention to selected tokens [65]. However, pruning strategies in HPE cannot rely solely on global response magnitude or parameter size. Regions with weak responses may still correspond to occluded joints, small-scale joints, or body parts near image boundaries. Without accounting for human body structure and keypoint importance, pruning may reduce the overall FLOPs while substantially degrading localization accuracy at specific joints. Quantization remains one of the most widely used optimization methods in practical deployment. Integer-only quantization has shown that low-bit integer inference can improve inference efficiency on mobile devices [82]. ONNX Runtime supports 8-bit linear quantization and graph optimization, TensorRT supports low-precision inference types such as INT8, and Core ML Tools also provides model quantization and compression pipelines [83,84,85,86]. HPE, however, is generally more sensitive to quantization errors than image classification tasks. Small logit perturbations in classification may not change the final category prediction, whereas slight shifts in heatmap peaks, coordinate decoding, or sub-pixel localization in HPE can directly introduce keypoint coordinate errors.

Lightweight optimization in HPE should be evaluated not only by parameter count, FLOPs, or model size but also by real-device latency, memory usage, quantization-induced changes in AP/MPJPE, power consumption, and temporal stability in video inference. In rehabilitation assessment, action scoring, and human–computer interaction, small drifts in local keypoints may have more practical impact than a reduction in overall AP. Real-world deployment usually requires lightweight architectures, distillation, pruning, quantization, and inference optimization frameworks to be combined rather than applied independently. Table 6 summarizes the major lightweight optimization strategies for deployable HPE, including their optimization targets, deployment benefits, potential risks to pose estimation accuracy, and suitable deployment platforms.

### 5.5. Deployment Trade-Offs and Method Selection

In real-world deployment, different method families reflect different trade-offs among accuracy, latency, memory usage, power consumption, temporal stability, hardware compatibility, and engineering maturity rather than interchangeable solutions. CNN-based methods benefit from mature convolution operators, stable deployment toolchains, and quantization-friendly architectures, making them suitable for edge devices, industrial vision systems, and real-time applications; their main limitations are weaker long-range dependency modeling and a reduced ability to capture complex spatial relationships. Transformer-based methods provide stronger global feature modeling and often achieve higher accuracy on large-scale benchmarks, especially in high-resolution or multi-person settings. These gains come with higher GPU memory consumption, increased attention complexity, stronger dependence on large-scale pretraining, and more difficult quantization and deployment. Temporal methods improve motion continuity and prediction stability in video-based HPE, but they introduce additional sequence computation, feature caching, and temporal latency. Mamba/SSM-based approaches may reduce the computational burden of long-sequence modeling, although their effectiveness and deployment behavior in HPE still require further validation.

**Figure 4:**
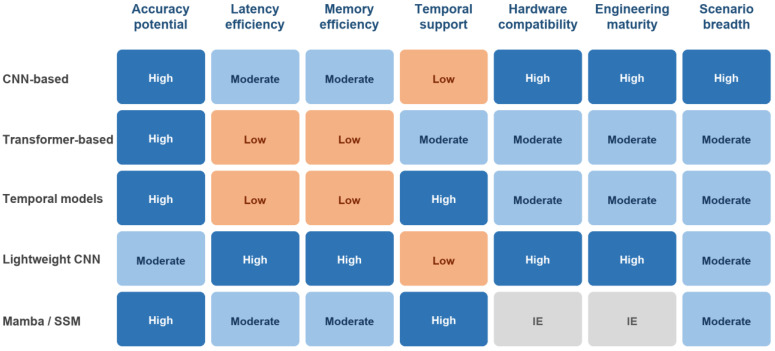
Qualitative deployment profile of major HPE method families. All criteria are oriented so that higher is more favorable. High indicates consistently favorable evidence among representative methods; moderate indicates conditional or mixed evidence; low indicates a recurring deployment constraint; IE indicates insufficient real-device evidence for a stable judgment. Ratings synthesize representative literature and Table 3, Table 4, Table 5, Table 6 and Table 7; they are an evidence-informed qualitative synthesis, not a meta-analysis.

Method selection in practical systems should be guided by deployment constraints rather than benchmark rank alone. High-capacity CNN or Transformer models can support precision-oriented offline analysis, whereas lightweight architectures and device-specific optimization are often better starting points for mobile or edge use. Temporal models are important when trajectory stability, 3D lifting, or motion understanding is required, but causality and buffering must match the response budget. Figure 4 summarizes the evidence-informed qualitative profile of major method families using criteria defined so that higher levels are consistently more favorable. Representative evidence includes CNN baselines [5,6,8,51,52,53,54,55,56,57,58], Transformer methods [7,59,60,61,62,63,64,65], temporal and state-space models [10,47,48,49,50,67,68,69,70,71,72,73,74,75,76,77,78], and lightweight or deployment-optimization studies [9,29,79,80,81,82,83,84,85,86,89], with method profiles and quantitative reporting summarized in Table 3, Table 4, Table 5, Table 6 and Table 7.

The main conclusion of this section is that HPE method selection is a constraint-matching problem rather than a benchmark-ranking exercise. High AP does not guarantee edge suitability, low FLOPs do not guarantee lower end-to-end latency, and stronger temporal modeling can add look-ahead or buffering cost. State-space models offer a promising route for long-sequence processing, but HPE-specific real-device evidence, quantization behavior, operator support, and complete-pipeline timing remain limited. Revised Table 7 therefore focuses on reported accuracy, computation, runtime/hardware context, and temporal support while avoiding repetition of the qualitative deployment profiles in Table 3, Table 4 and Table 5. N/R is used whenever a complete-pipeline value is absent or not directly comparable.

## 6. Application Scenarios and Reliability Requirements

Benchmark AP is the wrong evaluation criterion for deployment decisions. A model that satisfies an offline annotation pipeline—where latency is unconstrained, inputs are preprocessed, and errors affect only a label file—fails categorically in rehabilitation feedback, pedestrian intention estimation, or industrial safety monitoring, where temporal stability, response delay, and joint-level error sensitivity directly determine whether the system is safe to operate [1,37,40,90]. These are not stricter versions of the same requirement—they are different requirements, and no leaderboard score encodes them.

Application scenarios do not merely illustrate where HPE is used; they impose different evaluation priorities. Interactive systems require a response budget matched to the device, interaction loop, and user task rather than a universal frame-period threshold. Rehabilitation emphasizes repeatability, joint-angle bias, and stability across sessions; sports and long-duration monitoring emphasize trajectory quality and identity consistency; autonomous and industrial systems emphasize uncertainty, event-level errors, and downstream risk. Because pose is usually an intermediate representation for recognition, scoring, or decision logic, method selection and evaluation must be tied to the complete application pipeline.

### 6.1. Sports Analysis and Training

In sports training and competitive analysis, HPE is used for motion technique evaluation, performance optimization, injury-risk assessment, and biomechanical analysis rather than simple skeleton visualization [38,91,92]. These systems commonly process continuous video captured by fixed, mobile, or multi-view cameras, often with high frame rates or synchronized multi-view acquisition. Offline or near-real-time processing may be acceptable in some scenarios, but temporal stability remains critical because frame-level jitter can distort estimates of speed, rhythm, joint angle, and motion-cycle timing.

The primary deployment challenge in sports applications is maintaining temporal and biomechanical consistency across continuous motion sequences. A model may achieve high per-frame joint accuracy yet remain unsuitable for fast sports movements if limb trajectories fluctuate over time. In training feedback systems, local errors at the knee, elbow, shoulder, or ankle can alter the interpretation of movement quality or technique execution. Competitive sports analysis introduces additional difficulties because multi-athlete interactions and camera motion increase the risk of tracking failure and identity switching.

A concrete sports example is continuous technique analysis from ordinary video: the output must preserve fast wrist, elbow, knee, and ankle trajectories while maintaining athlete identity through camera motion and interaction. Existing sports-oriented HPE studies illustrate that task value comes from stable kinematic measurements and action-specific interpretation, not from skeleton visualization alone [91,92].

### 6.2. Rehabilitation and Clinical Assessment

Rehabilitation and clinical assessment systems commonly use RGB cameras, mobile-phone videos, RGB-D cameras, or wearable sensing devices to capture human movement. The objective is not skeleton visualization itself but the extraction of clinically relevant measurements such as range of motion, gait characteristics, movement quality, and recovery progression [37,39,93,94,95]. Although these applications do not always require very high frame rates, they remain sensitive to local joint errors and temporal discontinuities because small keypoint deviations can alter clinical measurements and movement interpretation.

Rehabilitation scenarios place greater emphasis on measurement reliability, consistency across repeated sessions, and uncertainty awareness than general video analysis tasks. The system should remain stable under changes in camera placement, clothing, body shape, and mobility condition while also indicating when predictions become unreliable. Clinical HPE systems should therefore be evaluated not only by keypoint accuracy but also by agreement with clinically meaningful movement measurements and by stability across different patient populations.

Markerless and sensor-based rehabilitation systems therefore need evaluation against clinically meaningful measurements rather than relying only on visual plausibility or benchmark keypoint metrics [96,97].

In rehabilitation, camera-based range-of-motion assessment provides a practical case in which repeated-session agreement and joint-angle bias matter more than visually plausible keypoints. Studies of markerless rehabilitation and mobile assessment show the need to validate pose-derived measurements against clinical or sensor references and to report uncertainty when camera placement or patient mobility changes [39,94,95].

### 6.3. Human–Computer Interaction

In human–computer interaction, HPE supports gesture control, body-motion interaction, virtual character control, and real-time feedback [1,28,98]. Many HCI systems operate on mobile, embedded, or edge devices, where latency, power consumption, privacy, and sensitivity to user appearance, camera placement, and background variation directly affect usability. Even when average pose accuracy is high, delayed or unstable predictions can still degrade interaction quality.

The main deployment requirement in HCI is immediate and predictable system response. Users are often sensitive to delay, flicker, and inconsistent gesture recognition, even when the underlying pose error is relatively small. Lightweight models, causal smoothing, and device-specific optimization are therefore important for maintaining stable interaction under limited computing resources. Privacy constraints and low-latency requirements may also favor on-device inference over cloud-based high-capacity models. This HCI perspective is closely related to human activity recognition, where vision, mobile, and wearable sensors are used to convert human motion into interaction or decision signals [12,26].

BlazePose provides a representative HCI deployment case: it was designed for real-time full-body tracking on mobile devices and reports more than 30 frames per second on a Pixel 2 [28]. This example is useful because its relevance depends on the complete on-device pipeline and interaction stability, not only benchmark AP.

### 6.4. Autonomous Driving and Pedestrian Understanding

In autonomous driving, HPE is typically used as an intermediate cue for pedestrian intention understanding, action prediction, and risk assessment within a larger perception pipeline [40,41,42,43,99]. Input data are usually captured by vehicle-mounted cameras and may be combined with other sensing modalities. Long-distance observation, occlusion, weather variation, and motion blur substantially increase the data gap, while downstream tasks depend more on reliable behavior interpretation than on visually plausible skeleton reconstruction alone.

In this scenario, pose estimation results are usually interpreted together with tracking, scene context, trajectory prediction, and—in some systems—LiDAR or radar information. The main deployment concern is not whether every joint can be localized with high precision, but whether pose cues improve the reliability of pedestrian behavior understanding under safety-critical conditions. This increases the importance of uncertainty estimation, multimodal consistency, and failure detection, particularly when perception quality degrades because of occlusion, low visibility, or sensor disagreement.

HUM3DIL illustrates a deployment-oriented autonomous-driving case by combining image and LiDAR information for 3D human pose estimation [99]. The example reinforces that pose cues should be evaluated together with multimodal consistency, tracking, and downstream pedestrian understanding rather than as an isolated skeleton output.

### 6.5. Industrial Safety Monitoring

Industrial and construction safety monitoring systems commonly rely on fixed surveillance cameras or edge vision sensors for continuous observation. HPE can support fall detection, PPE compliance monitoring, worker behavior analysis, multi-worker tracking, and construction activity understanding [44,45,90,100,101,102]. These applications place greater emphasis on long-term online operation, low maintenance cost, tolerance to occlusion and protective clothing, and event-level recall. In many cases, the key requirement is whether the system can reliably support safety-related decisions, rather than whether it achieves the highest single-frame AP.

Industrial environments also introduce practical constraints that are rarely represented in benchmark datasets. Cameras may be installed at high viewing angles, workers can be partially occluded by equipment, and visual appearance may change because of helmets, safety vests, tools, dust, or illumination variation. Since these systems often operate continuously, both false alarms and missed events can produce operational and safety costs. As a result, deployment concerns extend beyond pose estimation accuracy to include calibration stability, long-term reliability, and maintainability.

Industrial PPE and construction-monitoring studies provide concrete examples of continuous fixed-camera deployment, where worker occlusion, elevated viewpoints, re-identification, and event-level false alarms determine usefulness [45,90,100,101,102]. Their lessons favor reporting uptime, event recall, identity continuity, and maintainability alongside keypoint accuracy.

### 6.6. Scenario-Driven Method Selection and Evaluation Priorities

The preceding scenarios suggest that deployment-oriented HPE should be selected according to application requirements rather than model categories alone. A practical deployment pipeline should first define the target scene, input source, latency budget, hardware platform, acceptable failure risk, and downstream task. Model selection then becomes a constraint-driven process involving trade-offs among high-accuracy CNN or Transformer backbones, lightweight real-time models, temporal models, and multi-stage tracking pipelines.

In sports analysis and rehabilitation, stable and repeatable motion measurement is often more critical than single-frame keypoint accuracy alone. These applications typically rely on accurate 2D backbones, temporal smoothing, 3D lifting, and careful camera calibration, particularly when joint angles, gait features, or range-of-motion measurements are used for clinical or biomechanical interpretation. Human–computer interaction and mobile applications place greater emphasis on low latency, response stability, and on-device efficiency. Under these constraints, compact CNNs, real-time pose estimators, quantization-friendly architectures, and causal smoothing are often more suitable than large offline models with higher computational cost.

Safety-critical applications impose different system priorities. In autonomous driving, pose estimation is usually not the final output but a single cue integrated with detection, tracking, trajectory prediction, scene context, and—in some systems—LiDAR or radar data. The main question is whether pose cues improve pedestrian behavior understanding under long-distance, occluded, or low-visibility conditions. Industrial and construction monitoring systems must operate continuously under fixed-camera settings, equipment occlusion, protective clothing, and changing illumination. In these environments, event-level recall, false-alarm cost, and long-term maintainability may be more important than single-frame AP.

These differences indicate that no single HPE model is suitable for all real-world deployment scenarios. A model designed for cloud-based sports analysis may not satisfy the latency requirements of interactive feedback systems. Lightweight mobile models may be insufficient for clinical measurement tasks that require stable and precise motion estimation, while highly accurate single-frame models may still produce unstable predictions during long-term monitoring. Method evaluation should therefore include not only accuracy but also latency, memory consumption, temporal stability, robustness under expected visual conditions, and the effects of pose estimation errors on downstream decisions.

Scenario-driven method selection requires matching the model family to the dominant deployment constraint. Accuracy-oriented backbones are more suitable when sufficient computational resources are available and measurement precision is a primary requirement. Lightweight and quantization-friendly models are preferable for applications constrained by latency, power consumption, or edge-device deployment. Temporal models are useful when temporal stability, 3D lifting, or motion interpretation is required, although their buffering overhead and inference delay should also be evaluated. In safety-critical or decision-oriented applications, uncertainty estimation and failure-mode reporting are important because the impact of an error depends on the application outcome, not only on the keypoint error itself. Table 8 summarizes the requirement characteristics of different scenarios and the corresponding trends in method selection.

The five scenarios activate the four deployment gaps in different combinations. Table 9 records the dominant gaps, the mechanism that makes them important, and the indicators that should receive priority. The table intentionally avoids unsupported high/medium/low scores; the stated priorities are application constraints to be validated for the actual system.

Although the five application scenarios impose different evaluation priorities, the evidence required to assess deployment readiness shares a common core. To improve reproducibility and cross-study comparison, Table 10 consolidates the minimum information that deployment-oriented HPE studies should report. Scenario-specific measures may be added, but the sensing conditions, pipeline boundary, hardware and software configuration, latency definition, resource use, robustness, temporal behavior, and task-level outputs should be stated explicitly.

## 7. Conclusions and Outlook

Real-world human pose estimation should not be viewed solely as a problem of improving keypoint localization accuracy under controlled benchmark conditions. Public datasets and standard metrics such as AP, PCK, AUC, and MPJPE remain essential for method development and comparison [2,3,4,15], but they provide only partial evidence of deployment readiness. In practical systems, pose estimation is embedded within a longer sensor-to-decision pipeline that includes image acquisition, detection, cropping, pose inference, coordinate decoding, tracking, smoothing, and task-specific interpretation. Errors introduced at any stage can propagate through the pipeline and affect downstream decisions. The central question is therefore not only which model achieves the highest benchmark score, but whether the complete system remains reliable under the visual, computational, temporal, and application constraints of the target scenario.

This review argues that the deployment gap in HPE arises from four interacting factors: data conditions, system constraints, task requirements, and temporal behavior. Data gaps emerge when models trained on curated datasets encounter occlusion, low resolution, motion blur, lighting variation, crowding, unusual camera viewpoints, or domain shift in real environments [16,17,18,19,20]. System gaps arise because deployed applications must satisfy hardware, memory, latency, power, and runtime-framework constraints in addition to pose accuracy. Task gaps appear when application performance depends on joint-angle estimation, gesture response, pedestrian-risk assessment, fall detection, or safety-event recognition rather than visually plausible keypoints alone. Temporal gaps arise when frame-level predictions must be transformed into stable trajectories, identity-consistent tracks, and temporally smooth downstream signals [47,48,49]; these gaps interact during deployment. Occlusion may first reduce pose confidence, tracking errors may then propagate the failure across frames, and a small keypoint deviation may ultimately produce an incorrect clinical measurement or a missed safety event.

Future evaluation protocols should become more condition-aware and more transparent. Instead of reporting only average benchmark performance, deployment-oriented studies should stratify results by person scale, occlusion level, lighting condition, camera viewpoint, motion speed, crowd density, and input resolution. Video-based HPE evaluation should also include trajectory stability, temporal jitter, identity consistency, recovery after missed detections, and the latency introduced by smoothing or temporal modeling. For efficient HPE systems, reporting only FLOPs or parameter counts is insufficient. Studies should additionally report end-to-end latency, memory consumption, throughput, power consumption, precision mode, inference framework, hardware backend, preprocessing overhead, and post-processing overhead. These metrics provide a clearer indication of whether a method that performs well on a leaderboard can satisfy the constraints of mobile devices, embedded GPUs, browser-based runtimes, clinical workflows, vehicle perception systems, or industrial monitoring platforms.

Model design should also be evaluated in the context of deployment conditions. CNN-based methods benefit from mature operators and production toolchains [5,6,9], but theoretical complexity and operator maturity still do not guarantee lower complete-pipeline latency on a particular device; preprocessing, memory traffic, precision support, and backend mapping must be measured. Transformer-based methods can improve global structural modeling but often place greater demands on memory and attention kernels [7,59,60,61,62,63,64,65]. Temporal models are important for trajectory stability, 3D lifting, and motion understanding, although causality, window length, buffering, and inference latency must be reported [47,67,68,69,70,71,72]. Mamba and related state-space models are promising for long sequences, but their practical HPE value still requires task-specific benchmarks and real-device validation [10,73,74,75,76,77,78]. Lightweight architectures, pruning, quantization, distillation, ONNX Runtime, TensorRT, and Core ML can improve deployability; their effects should be evaluated on coordinate bias, joint-angle error, temporal jitter, and downstream task performance as well as AP or MPJPE [79,80,81,82,83,84,85,86,89].

Application-level validation should become a central part of future HPE evaluation. In sports analysis, models should be evaluated by whether they preserve motion trajectories and biomechanical indicators during fast movements and self-occlusion [38,91,92]. In rehabilitation scenarios, session-to-session repeatability, joint-angle bias, and measurement reliability may matter more than visually smooth skeleton predictions [37,39,93,94,95,96,97]. In human–computer interaction, latency, temporal jitter, and perceived response stability often affect usability more directly than absolute keypoint precision [28,98]. In autonomous driving, pose cues are useful only when they improve pedestrian intention estimation, risk prediction, or multimodal scene understanding under safety-critical conditions [40,41,42,43,99]. In industrial and construction monitoring, event-level recall, false-alarm cost, robustness to protective equipment, and long-term maintainability are often more informative than single-frame AP [44,45,90,100,101,102]. These scenarios illustrate that pose estimation errors should be evaluated according to their impact on downstream decisions, measurements, and user experience.

The field is gradually shifting from model-centered evaluation toward deployment-centered design. This shift does not reduce the importance of architectural innovation but places model architecture within a broader system context. Future HPE methods will likely be more useful when they explicitly state their operating assumptions, report performance under realistic deployment constraints, and demonstrate that pose outputs remain meaningful for the target application. A deployable HPE system should not only achieve high accuracy but also maintain robustness, computational efficiency, temporal stability, interpretability, and long-term maintainability under practical conditions. Progress in real-world HPE will therefore depend less on a single universal architecture and more on the alignment between data conditions, system resources, temporal requirements, and application-level reliability.

## Figures and Tables

**Table 1:** Positioning of the present review relative to recent HPE surveys.

Review	Main Scope	Temporal/Tracking	Deployment/System Metrics	Application Reliability	Distinctive Emphasis
Zheng et al. (2024) [1]	Broad deep-learning HPE taxonomy	Covered	Limited	General applications	Methods, datasets, and challenges
Nogueira et al. (2025) [14]	Markerless multi-view 3D HPE	Central	Complexity discussed	Multi-view use cases	Multi-view and multimodal 3D estimation
Chen et al. (2025) [21]	Monocular video HPE and tracking	Central	Real-time aspects	Video applications	Video-based estimation and tracking
Dibenedetto et al. (2025) [22]	2D/3D deep-learning HPE	Covered	Performance overview	Broad applications	Techniques, datasets, and frameworks
Kappan et al. (2026) [23]	2D/3D, image/video HPE	Covered	Challenges discussed	Broad applications	Comprehensive field survey with explicit selection process
Zhang and Shin (2025) [24]	Deep-learning 2D HPE	Limited	Efficiency as a method property	General applications	Method taxonomy, metrics, datasets, and benchmark leaders
Present review	Sensor-to-decision deployment of HPE	Deployment gap and stability	End-to-end latency, memory, power, backend, and reporting boundaries	Scenario-specific risk and task outputs	Four-gap diagnosis, full pipeline, application mapping, and reporting checklist

**Table 2:** Pipeline modules, bottlenecks, risks, and mitigation strategies.

Pipeline Module	Input/Output	Main Bottleneck	Failure Propagation	Relevant Metrics	Mitigation Strategy
Sensor image/ video acquisition	Camera, RGB-D, or video sensor stream	Noise, blur, lighting, viewpoint, compression	Low-quality frames reduce detector and pose confidence	Resolution, FPS, blur, illumination, frame drop	Sensor calibration; quality filtering; domain adaptation
Human detection	Frame/person boxes	Missed persons, false positives, detector latency	Misses cannot be recovered by pose estimator	Recall, precision, latency, crowded-scene robustness	Robust detector; confidence gating; scene-specific tuning
Region localization	Boxes/normalized crops	Crop shift, scale error, resizing artifacts	Keypoints shift or disappear near crop boundaries	Box IoU, crop consistency, scale sensitivity	Box expansion; tracking-assisted boxes; multi-scale input
Pose inference	Crop/features/keypoints or heatmaps	Compute, memory, output encoding, coordinate decoding	Local errors affect measurement and downstream decisions	AP, PCK, AUC, MPJPE, latency, memory	Efficient backbone; robust decoding; uncertainty estimation
Tracking/association	Frame poses/identities	ID switch, fragmentation, occlusion recovery	Broken trajectories corrupt temporal features	IDF1, MOTA, track continuity, recovery rate	Appearance-motion association; pose-aware tracking
Smoothing/transform	Pose tracks/stable coordinates	Window latency, calibration, over-smoothing	Delayed or biased motion signal	Jitter, lag, smoothness, coordinate error	Causal filters; adaptive windows; camera calibration

**Table 3:** Deployment-oriented profile of representative CNN-based HPE methods.

Method/Representative Work	Deployment Role	Deployment Advantages	Deployment Cost Driver	Deployment Limitations	Optimization Leverage	Suitable Deployment Scenario
DeepPose [51]	Early coordinate-regression baseline; historical reference for direct pose prediction.	Simple end-to-end formulation; avoids explicit heatmap storage and decoding.	Fully connected regression head; limited representation compared with later CNN pipelines.	Lower localization precision; weak spatial interpretability; not a modern deployment baseline.	Direct coordinate heads; compact regression output; reference for heatmap-free designs.	Historical comparison; lightweight proof-of-concept; not preferred for high-reliability deployment.
Simple Baseline [5]	Strong top-down CNN baseline after human detection.	Mature ResNet backbone; easy reproduction; convolution operators are runtime- and quantization-friendly.	Detector dependency; per-person crop inference; deconvolution head and heatmap resolution.	Latency scales with person count; crop errors propagate to keypoints; limited temporal stability.	Backbone scaling; input-size reduction; INT8/FP16 inference; batched crop processing.	Server or edge GPU top-down systems with moderate person count and stable detector quality.
CPN [52]	Multi-stage refinement CNN for difficult keypoints in top-down HPE.	Improves hard keypoint localization; refinement modules target occlusion-prone joints.	Cascaded stages; global and refine networks; larger memory and inference cost.	Less attractive for strict real-time deployment; multi-stage design increases engineering complexity.	Selective refinement; stage pruning; deploy refinement only for uncertain joints.	Offline analysis or accuracy-prioritized systems where hard-joint recovery matters more than latency.
HRNet-W32/W48 [6]	High-accuracy CNN backbone maintaining high-resolution representations.	Strong localization accuracy; mature convolutional toolchain; stable benchmark performance.	Parallel high-resolution branches; feature-map memory traffic; W48 increases compute and memory cost.	Heavy for low-power edge devices; high-resolution features may dominate memory bandwidth.	Choose W32 over W48 for deployment; mixed precision; channel pruning; resolution-aware inference.	High-end edge GPU or server-side pose estimation requiring accurate 2D keypoint localization.
HigherHRNet [53]	Bottom-up high-resolution CNN for multi-person scenes.	Avoids repeated single-person pose inference; useful when many people appear in one frame.	High-resolution heatmaps; keypoint grouping; multi-scale testing can be expensive.	Grouping errors in crowded scenes; memory-heavy heatmaps; cost depends on image scale.	Single-scale deployment; efficient grouping; heatmap resolution control; crowd-aware post-processing.	Crowded multi-person scenes where top-down per-person inference becomes costly.
Lite-HRNet [9]	Lightweight high-resolution CNN for efficient pose estimation.	Small parameter count; low GFLOPs; preserves high-resolution representation with better edge suitability.	Lightweight blocks and channel weighting; accuracy still depends on input resolution and training protocol.	May underperform heavier HRNet under severe occlusion or high-precision requirements.	Lightweight high-resolution design; mobile/edge acceleration; quantization-aware validation.	Compute-limited edge devices, mobile HPE, industrial cameras, and real-time feedback systems.

**Table 4:** Deployment-oriented profile of Transformer-based HPE methods.

Method/Representative Work	Deployment Role	Deployment Strength	Deployment Bottleneck	Pretraining/Runtime Dependence	Optimization Leverage	Recommended Scenario
TransPose [61]	Early Transformer-style keypoint localization using attention for structural relationships.	Better long-range dependency modeling than pure local convolution.	Attention cost and memory grow with token count; limited real-device deployment evidence.	Requires mature Transformer kernels; benefits from strong visual pretraining.	Token reduction; hybrid CNN–Transformer design; lower input resolution.	Research prototype or offline analysis where structural modeling is more important than latency.
TokenPose [62]	Keypoint-token representation for explicit joint modeling.	Interpretable keypoint tokens; captures joint relations directly.	Token interaction cost; not primarily designed for low-power deployment.	Depends on Transformer training stability and token design.	Reduce keypoint/image tokens; lightweight attention; distillation to CNN or compact Transformer.	High-precision offline 2D HPE and scenarios needing explicit joint-token representation.
HRFormer [64]	High-resolution Transformer family for dense prediction and HPE-related representation.	Combines high-resolution representation with stronger global modeling.	High-resolution attention blocks raise memory traffic and operator complexity.	Backend support for window/attention operators affects real latency.	Windowed attention; mixed precision; branch/channel reduction.	Cloud or high-end GPU deployment requiring high-resolution structural features.
ViTPose-B/L [7]	High-capacity Transformer baseline for top-down 2D HPE.	Strong AP; scalable model family; simple pipeline when compute is available.	Large variants are unsuitable for ordinary edge devices; attention and large hidden states increase memory.	Strongly benefits from MAE/large-scale pretraining; runtime optimized for GPU/accelerator rather than CPU.	Use smaller variants; distillation; token pruning; FP16 inference; server-side batching.	Cloud inference, high-end edge GPU, offline sports/clinical analysis requiring high accuracy.
PPT [65]	Token-pruned pose Transformer for reducing token redundancy.	Explicitly targets Transformer efficiency by pruning tokens.	Pruning policy adds control complexity; accuracy can depend on retained token quality.	Needs compatible implementation for dynamic or static pruning.	Static token pruning; deployment-aware pruning ratio; benchmark on target hardware.	Transformer deployment when memory/latency must be reduced without fully abandoning attention.
RTMPose [8]	Real-time engineering-oriented HPE family in MMPose.	Strong speed–accuracy balance; practical CPU/GPU/mobile deployment reports; compact variants available.	Still depends on preprocessing, detector, and backend-specific optimization.	Benefits from MMPose/MMDeploy ecosystem and optimized runtime kernels.	TensorRT/ONNX deployment; input-size tuning; lightweight model selection; detector–pose co-design.	Real-time production-like systems, mobile/edge HPE, industrial feedback, and interactive applications.

**Table 5:** Deployment-oriented comparison of temporal modeling methods for video HPE.

Temporal Method	Deployment Motivation	Complexity/Latency Pattern	Strength	Deployment Limitations	Optimization Leverage	Best-Fit Scenario
VideoPose3D/TCN [47]	Reduces single-frame jitter and supports 2D-to-3D lifting with temporal context.	Temporal convolution; cost grows with receptive field but remains more deployment-friendly than full attention.	Lightweight relative to long-window Transformers; stable and simple to implement.	Limited long-range context; large receptive field may require wider windows and buffering.	Causal convolution; dilated TCN; smaller temporal window; streaming cache.	Online or near-real-time 3D lifting where latency budget is limited.
PoseFormer [67]	Uses spatiotemporal Transformer modeling for stronger sequence representation.	Self-attention cost can grow quadratically with temporal tokens; windowing adds latency.	Captures global temporal dependencies and complex motion patterns.	Not ideal for low-latency edge deployment; future-frame context may break real-time use.	Shorter windows; causal attention; token reduction; offline batching.	Offline sports analysis, clinical review, and video reconstruction where future frames are available.
MHFormer [68]	Models multiple pose hypotheses to improve ambiguous 3D reconstruction.	Multiple hypotheses increase compute and memory compared with single-branch temporal models.	Robust to ambiguity and uncertainty in 2D-to-3D lifting.	Heavier inference; more difficult to compress without losing uncertainty coverage.	Reduce hypothesis count; distill to single student; deploy only when ambiguity is high.	Offline 3D pose estimation under ambiguous views or occlusion.
MixSTE [69]	Separates and mixes spatial–temporal encoding for high-accuracy video-based 3D HPE.	Long-sequence modeling; reported high FLOPs/parameter cost under common Human3.6M protocols.	Strong temporal–spatial representation and competitive MPJPE.	High compute makes real-time edge deployment difficult.	Sequence length reduction; temporal sampling; distillation; low-rank or sparse attention.	Cloud/offline motion analysis where accuracy outweighs latency.
PoseFormerV2 [71]	Improves efficiency by exploring frequency-domain temporal representation.	Aims to reduce redundancy in long temporal sequences.	Better efficiency direction than plain temporal attention for long sequences.	Still needs careful protocol-specific validation and optimized implementation.	Frequency-domain compression; shorter local windows; streaming-compatible design.	Efficient offline or near-real-time 3D HPE with moderate sequence length.
MotionBERT [72]	Uses motion representation pretraining for multiple motion understanding tasks.	Pretraining and adaptation cost are high; inference depends on sequence length and downstream head.	Strong transferable motion prior; useful beyond pose estimation alone.	Large pretraining pipeline; deployment requires task-specific fine-tuning and validation.	Task-specific head pruning; sequence caching; distillation; uncertainty-aware deployment.	Cloud analytics, rehabilitation/sports assessment, and multi-task human motion understanding.
Mamba/SSM family [73,74,75,76,77,78] and PoseMamba [10]	Targets long-sequence modeling with linear-time state-space mechanisms.	Linear-time sequence processing is attractive for long video streams.	Potentially better long-stream efficiency than O(n^2) temporal attention.	HPE-specific deployment evidence and runtime/toolchain maturity remain limited.	Streaming state reuse; hybrid local–global modeling; benchmark on real devices before deployment claims.	Emerging research direction for long-video HPE and deployment-aware temporal modeling.

**Table 6:** Lightweight optimization strategies for deployable HPE systems.

Strategy	HPE-Specific Target	Deployment Benefit	HPE-Specific Deployment Risk	Implementation Considerations	Suitable Deployment Platform
Knowledge distillation [89]	Transfer structural pose knowledge from a high-capacity teacher to a compact student.	Improves lightweight model accuracy without increasing inference cost.	Student may inherit teacher bias; weak distillation can miss local joints or occluded parts.	Use joint-level, heatmap-level, and structure-aware losses; validate on hard joints.	Mobile/edge models that need teacher-level accuracy at lower cost.
Structured/channel pruning	Remove redundant channels, blocks, or branches while preserving keypoint-sensitive features.	Reduces parameters, FLOPs, and memory bandwidth.	Pruning may damage small-joint localization or body-part balance even if AP drops slightly.	Prefer structured pruning; tune per-stage pruning ratio; retrain and check joint-specific error.	CNN backbones on GPU, CPU, NPU, or embedded accelerators.
INT8 quantization [82]	Low-precision inference for backbone, head, and sometimes post-processing.	Lower memory footprint; higher throughput; better power efficiency.	Heatmap peak shifts, coordinate drift, and sub-pixel errors can directly move keypoints.	Use calibration data covering poses, lighting, scales, and occlusion; consider QAT for sensitive heads.	TensorRT, ONNX Runtime, Core ML, mobile/edge NPUs.
ONNX Runtime optimization [83,84]	Graph optimization, operator fusion, quantization, and backend execution planning.	Improves portability and backend-agnostic acceleration.	Unsupported or decomposed operators may reduce speed; CPU–GPU transfer can dominate.	Export with static shapes when possible; profile complete pipeline, not only model forward.	Cross-platform CPU/GPU deployment and production inference services.
TensorRT optimization [85]	GPU inference acceleration with precision selection and kernel fusion.	High throughput and low latency on NVIDIA GPUs; strong FP16/INT8 support.	Accuracy may change after precision lowering; dynamic shape and custom ops require care.	Validate output numerically; build engines for target input shapes; calibrate INT8 carefully.	NVIDIA edge GPU, workstation GPU, cloud GPU inference.
Core ML/mobile runtime optimization [86]	Convert and compress models for Apple or mobile deployment.	On-device privacy, lower latency, and power-aware execution.	Operator coverage and quantization behavior can differ from PyTorch/ONNX results.	Check converted model outputs; validate camera input pipeline and preprocessing.	iPhone/iPad/macOS applications and mobile HCI.
Lightweight architecture co-design [9,29,79,80,81]	Design model family around target compute, memory, and latency constraints.	Better deployment gains than post hoc compression alone.	Over-specialized design may lose accuracy under occlusion or domain shift.	Co-design backbone, head, input size, and runtime; validate on target device.	Resource-constrained edge devices, mobile HPE, real-time industrial cameras.

**Table 7:** Quantitative and reporting-oriented comparison of representative HPE methods. N/R = not reported in the cited source or not directly comparable. FLOPs and MACs follow the original sources and should not be compared without considering input size and counting convention. Runtime entries distinguish model-specific reports from complete sensor-to-output latency; no cross-paper end-to-end ranking is inferred.

Method	Model Family	Evaluation Setting	Accuracy	Parameters/Computation	Reported Runtime/Hardware	Temporal Support
SimpleBaseline-R50 [5]	CNN, top-down	COCO val2017, 256 × 192	AP 70.4	34.0M; 8.9 GFLOPs	N/R for complete pipeline	No
HRNet-W32 [6]	CNN, high-resolution	COCO val2017, 256 × 192	AP 74.4	28.5M; 7.1 GFLOPs	N/R for complete pipeline	No
ViTPose-B [7]	Transformer	MS COCO val, 256 × 192, MAE pretraining	AP 75.8	N/R	N/R for complete pipeline	No
RTMPose-m [8]	Real-time HPE	MMPose Body8/COCO report, 256 × 192	COCO AP 74.6	13.59 M; 1.93 GFLOPs	Backend/device- specific results reported; complete pipeline N/R	No
Lite-HRNet-18 [9]	Lightweight CNN	COCO val2017, 256 × 192	AP 64.8	1.1 M; 205.2 M FLOPs	N/R for complete pipeline	No
EfficientPose [29]	Efficient CNN/NAS	Lightweight HPE family	N/R	N/R	N/R	No
LitePose-S [81]	Edge multi-person	COCO val2017	mAP 56.8	N/R parameters; 5.0 G MACs	N/R for complete pipeline	No
PoseFormer [67]/MotionBERT [72]	Temporal/3D Transformer	Protocol-dependent video benchmarks	Protocol-dependent	N/R	N/R for complete sensor-to-output pipeline	Yes
PoseMamba/SSM family [10,73,74]	State-space/Mamba	Emerging image/video sequence modeling	Task-dependent	N/R	Insufficient HPE-specific real-device evidence	Yes

**Table 8:** Application scenarios and corresponding HPE method selection trends.

Scenario	Typical Input	Accuracy Requirement	Real-Time Requirement	Major Deployment Challenge	Current Practical Solution	Mature Applicable Methods	Recommended Method Tendency
Sports analysis and training	High-frame-rate RGB video; multi-view cameras; competition or training footage	High local joint precision; stable limb angles; accurate motion trajectory	Offline or near-real-time; real-time needed for live feedback	Fast motion; motion blur; self-occlusion; multi-athlete tracking	Video-based pose estimation with tracking and temporal smoothing	HRNet [6], RTMPose [8], ViTPose [7], VideoPose3D [47], MixSTE [69]	Accurate 2D backbone + temporal smoothing; cloud/offline temporal models for detailed biomechanical analysis
Rehabilitation and clinical assessment	RGB camera; mobile-phone video; possible depth camera; repeated home/clinic sessions	Very high measurement reliability; repeatability; low bias in joint angles and range of motion	Usually low to moderate; interactive feedback may require near-real-time	Small keypoint errors affect clinical indicators; camera placement variation; patient diversity	Controlled camera setup; calibrated measurement; uncertainty-aware output	HRNet [6], RTMPose [8], Lite-HRNet [9], VideoPose3D [47], MotionBERT [72]	Stable and interpretable models; prefer robust CNN/real-time models with uncertainty estimation and repeated-session validation
Human–computer interaction	Mobile camera; webcam; embedded camera; edge device stream	Moderate pose accuracy; high response consistency	Very high; low latency is critical	Delay, jitter, unstable gestures, limited compute and power	On-device lightweight HPE with causal smoothing	RTMPose [8], Lite-HRNet [9], LitePose [81], EfficientPose [29]	Lightweight real-time models; quantization-friendly CNNs; causal temporal smoothing; local inference preferred
Autonomous driving and pedestrian understanding	Vehicle-mounted RGB cameras; possible LiDAR/radar fusion; long-distance pedestrian views	Pose need not be anatomically perfect; reliability for intention/action cues is more important	High; must fit real-time perception stack	Small pedestrians; occlusion; weather; night scenes; safety-critical false negatives	Pose as auxiliary cue combined with detection, tracking, trajectory prediction, and scene context	RTMPose [8], HRNet [6], ViTPose [7], tracking-based pipelines [31,32,33]	Robust detection–tracking–pose pipeline; uncertainty-aware pose cues; multimodal fusion when available
Industrial safety monitoring	Fixed surveillance cameras; wide-angle cameras; long-term video streams	Event-level reliability; high recall for unsafe actions or falls	Moderate to high; continuous online monitoring	Occlusion by tools/equipment; PPE; high camera angle; lighting changes; false alarms	Fixed-camera monitoring with tracking, action recognition, and alert logic	RTMPose [8], Lite-HRNet [9], HigherHRNet [53], OpenPose-style bottom-up methods [30], tracking-based pipelines [31,32,33]	Robust multi-person tracking; lightweight or bottom-up methods for crowded scenes; event-level validation over frame-level AP

**Table 9:** Dominant deployment gaps and evaluation priorities across application scenarios.

Scenario	Dominant Deployment Gap(s)	Why the Gaps Dominate	Priority Indicators
Sports analysis	Data; temporal; task	Fast limb motion, camera motion, athlete interaction, and kinematic interpretation	Trajectory jitter; joint-angle error; identity consistency; cycle/technique output
Rehabilitation	Task; temporal; data	Repeated sessions, patient/camera variability, and clinically meaningful measurement	Repeatability; joint-angle bias; uncertainty; session stability
Human–computer interaction	System; temporal	Resource-constrained devices and response predictability	End-to-end latency; latency jitter; power; gesture/task success
Autonomous driving	Data; system; task	Distance, occlusion, weather, multimodal fusion, and downstream safety risk	Track recovery; uncertainty; multimodal consistency; risk-prediction effect
Industrial safety	Data; system; task; temporal	PPE, equipment occlusion, continuous operation, alarms, and maintenance	Event recall; false-alarm rate; identity continuity; uptime; maintainability

**Table 10:** Minimum reporting checklist for deployment-oriented HPE studies.

Category	Minimum Information to Report
Sensor and input	Sensor/camera type; resolution; frame rate; compression; viewpoint; synchronization; number of people
Complete pipeline	Enabled stages from acquisition through detection, crop/resize, pose, tracking/filtering, coordinate transform, and task output
Hardware and software	Device and accelerator; CPU/GPU/NPU; memory; operating system; framework/backend; model version; precision; batch size
Latency and throughput	Explicit start/end boundary; mean plus distribution or percentiles; warm-up; end-to-end latency; throughput; latency jitter
Resource use	Peak/steady memory; model size; power or energy when relevant; thermal or sustained-load behavior
Robustness	Performance stratified by scale, occlusion, lighting, viewpoint, motion, crowding, resolution, and domain shift
Temporal behavior	Causality; window/look-ahead; filter parameters; jitter; identity switches; recovery definition and recovery time
Task-level reliability	Application output and reference standard; uncertainty/failure detection; false alarms/misses; downstream task effect

## Data Availability

The data used to support the findings of this study are available from the corresponding author upon request.
